# Serum anion gap at admission as a predictor of mortality in the pediatric intensive care unit

**DOI:** 10.1038/s41598-017-01681-9

**Published:** 2017-05-03

**Authors:** Min Jung Kim, Yoon Hee Kim, In Suk Sol, Soo Yeon Kim, Jong Deok Kim, Ha Yan Kim, Kyung Won Kim, Myung Hyun Sohn, Kyu-Earn Kim

**Affiliations:** 1 Department of Pediatrics, Severance Hospital, Institute of Allergy, Brain Korea 21 PLUS Project for Medical Science, Yonsei University College of Medicine, Seoul, Korea; 2 Biostatistics Collaboration Unit, Yonsei University College of Medicine, Seoul, Korea

## Abstract

An accurate method to predict the mortality in the intensive care unit (ICU) patients has been required, especially in children. The aim of this study is to evaluate the value of serum anion gap (AG) for predicting mortality in pediatric ICU (PICU). We reviewed a data of 461 pediatric patients were collected on PICU admission. Corrected anion gap (cAG), the AG compensated for abnormal albumin levels, was significantly lower in survivors compared with nonsurvivors (*p* < 0.001). Multivariable logistic regression analysis identified the following variables as independent predictors of mortality; cAG (OR 1.110, 95% CI 1.06–1.17; *p* < 0.001), PIM3 [OR 7.583, 95% CI 1.81–31.78; *p* = 0.006], and PRISM III [OR 1.076, 95% CI 1.02–1.14; *p* = 0.008]. Comparing AUCs for mortality prediction, there were no statistically significant differences between cAG and other mortality prediction models; cAG 0.728, PIM2 0.779, PIM3 0.822, and PRISM III 0.808. The corporation of cAG to pre-existing mortality prediction models was significantly more accurate at predicting mortality than using any of these models alone. We concluded that cAG at ICU admission may be used to predict mortality in children, regardless of underlying etiology. And the incorporation of cAG to pre-existing mortality prediction models might improve predictability.

## Introduction

To improve the quality of intensive care unit (ICU) care, mortality prediction is important^1^. However, estimation of mortality is difficult in critically ill patients whose condition may deteriorate. There are some invasive methods to assess the status of patients, such as pulmonary artery wedge pressure measurement, but these take time to institute and have side effects, such as infection. Recently developed assessments based on physiologic variables have limitations related to the high proportion of missing data, because the variables required are not collected for all patients admitted to ICU or not required for direct patient management^1^. Therefore it is necessary to identify noninvasive, easy tools for mortality prediction in ICUs, especially for pediatric ICU (PICU) patients.

Anion gap (AG) is traditionally one of the most commonly used biomarkers. It is the simplest means of evaluating the acid-base status of patients, and is calculated from the difference between the measured concentration of serum cations and anions. It helps to identify the presence and causes of metabolic acidosis^2, 3^. In addition, calculation of an initial serum AG in adult patients admitted to ICU has been suggested as a sensitive and specific tool to predict prognosis or mortality^4–6^. It has been shown that patients with a high AG have increased admission rates to hospital, increased rates of admission to ICU, and increased severity of illness, independent of concomitant electrolyte abnormalities^4^. An elevated AG is also associated with increased in-hospital mortality compared with patients having a normal AG^5, 6^. However, there is lack of data related to pediatric patients. In pursuit of better mortality prediction and proper management of patients in PICU, research on AG as a mortality predictor is meaningful.

In this study, we researched several factors associated with mortality in children admitted to PICU. Specifically, we investigated whether serum AG measured at the admission to PICU could be strong predictor of mortality, and what its predictive value is compared with other mortality prediction models.

## Results

### Patients’ characteristics

From December 2009 to February 2015, a total of 461 pediatric patients were included in this study. Predisposing medical diseases were neurologic problems (222 patients, 48.2%), such as intractable epilepsy or status epilepticus; respiratory problems (82 patients, 17.8%), such as respiratory infection or airway obstruction; and hematologic-oncologic problems (69 patients, 15%), such as neutropenic fever or tumor lysis syndrome. The patients’ baseline characteristics are presented in Table 1. The median age of the patients was 3.1 years and 58.1% (268/461) were male. Overall in-hospital mortality was 20.2% (93/461) with a median lengh of stay (LOS) in PICU of 10 days. A total of 349 children (75.7%) required mechanical ventilation within 24 hours of PICU admission. Overall, 281 (61%) patients had a metabolic acidosis. The most common reason for PICU admission was respiratory failure (51.6%).

**Table 1:** Clinical characteristics of patients in PICU.

	All (n = 461)
Age, years	3.1 (1.1–7.9)
Male, n (%)	268 (58.1)
LOS in PICU, days	10 (5–20)
In-hospital mortality, n (%)	93 (20.2)
Requirement for mechanical ventilation^a^, n (%)	349 (75.7)
Metabolic acidosis, n (%)	281 (61.0)
Reasons for PICU admission, n (%)	
Respiratory failure	238 (51.6)
Neurologic problem	72 (15.6)
Sepsis	40 (8.7)
Intensive monitoring	38 (8.2)
Post-resuscitation	37 (8.0)
Renal failure	32 (6.9)

Data are presented as number of patients (%) or median value (inter-quartile range).

PICU, pediatric intensive care unit; LOS, length of stay.

^a^Requirement for mechanical ventilation within 24 hours of PICU admission.

### Clinical Characteristics of survivors and non-survivors

Patients were divided into survivors and non-survivors on the basis of in-hospital mortality (Table 2). Survivors were younger than non-survivors were (median age, 2.4 vs. 5.8 years). Median LOS in PICU was statistically different between survivors and non-survivors, 9 days and 14 days, respectively. Non-survivors tended to require mechanical ventilation within 24 hours of PICU admission. The two groups were distinct in their reasons for PICU admission. The percentage of children with respiratory failure, neurologic problems, and requiring intensive monitoring was higher in survivors than in non-survivors. Meanwhile, a higher proportion of non-survivor had sepsis, post-resuscitation status, and renal failure. Scoring measurements for mortality prediction used in PICU (PIM2, PIM3, and PRISM III) were statistically different between survivors and non-survivors (*p* < 0.0001).

**Table 2:** Comparison between survivors and non-survivors.

	Survivors(n = 368)	Non-survivors(n = 93)	*p*-value
Age, years	2.4 (1.0–7.2)	5.8 (2.0–9.8)	0.0003
Male, n (%)	214 (58.2)	54 (58.1)	1.0000
LOS, days	9 (5–18)	14 (6–26)	0.0105
Requirement for mechanical ventilation^a^, n (%)	268 (72.8)	81 (87.1)	0.0042
Reasons for PICU admission, n (%)			
Respiratory failure	206 (56.0)	32 (34.4)	0.0003
Neurologic problem	62 (17.4)	8 (8.6)	0.0379
Sepsis	23 (6.2)	17 (18.3)	0.0007
Intensive monitoring	35 (9.5)	3 (3.2)	0.0563
Post-resuscitation	16 (4.3)	21 (22.6)	<0.0001
Renal failure	21 (5.7)	11 (11.8)	0.0639
Mortality prediction models in PICU			
PIM2 (logit_probability)	−0.99 (−1.25–−0.48)	−0.26 (−0.58–0.26)	<0.0001
PIM3 (logit_probability)	−1.15 (−1.40–−0.77)	−0.43 (−0.74–0.23)	<0.0001
PRISM III	5 (2–9)	16 (8–24)	<0.0001
Acid-base variables			
Metabolic acidosis, n (%)	221 (60.1)	60 (64.5)	0.3420
pH	7.40 (7.34–7.44)	7.36 (7.25–7.42)	0.0011
SBE, mEq/L	−3.39 (−6.74–0.37)	−5.26 (−10.4–−0.38)	0.0010
HCO_3,_ mEq/L	22.6 (19.4–26.6)	21.4 (16.5–25.6)	0.0367
Lactate, mmol/L	1.4 (0.9–2.3)	4.4 (1.6–11.1)	<0.0001
Sodium, mEq/L	139 (136–141)	140 (137–146)	0.0011
Albumin, g/dL	3.4 (3.0–3.8)	3.2 (2.9–3.6)	0.0087
AG, mEq/L	13.1 (9.8–15.5)	16.8 (12.5–22.5)	<0.0001
cAG, mEq/L	13.3 (10.1–16.0)	16.9 (12.8–23.0)	<0.0001
Biochemical values			
WBC, 10^3^/μL	11.2 (6.9–17.1)	10.9 (5.4–19.4)	0.6825
DNI%	0.6 (0.0–3.6)	4.2 (0.5–11.4)	<0.0001
Hb, g/dL	10.6 (9.4–11.7)	10.0 (8.2–11.2)	0.0217
Platelets, 10^3^/mm^3^	305 (176–448)	192 (87–361)	<0.0001
PTT, sec	32.2 (28.3–37.3)	37.6 (29.9–53.6)	<0.0001
D-dimer, ng/mL	588 (299–1144)	1788 (554–4998)	<0.0001
Glucose	126 (123–161)	151 (111–204)	0.0017
CRP, mg/L	7.8 (1.7–35.5)	20.4 (7.2–85.0)	<0.0001

Data are presented as number of patients (%) or median value (inter-quartile range).

LOS, length of stay; PICU, pediatric intensive care unit; PIM, Pediatric Index of Mortality; PRISM III, Pediatric Risk of Mortality III; SBE, standard base excess; AG, anion gap; cAG, corrected anion gap; WBC, white blood cell count; DNI, delta neutrophil index; Hb, hemoglobin; PTT, partial thrombin time; CRP, C-reactive protein.

^a^Requirement for mechanical ventilation within 24 hours of PICU admission.

More than 60% of patients in both groups had metabolic acidosis, but this had no statistical meaning. According to the summary data for measured and calculated variables, non-survivors had higher levels of sodium and lactate and lower levels of albumin, pH, SBE, and HCO3, compared with survivors. Both AG and cAG at admission were significantly higher in non-survivors than in survivors (*p* < 0.0001). There were significant differences in the median values of delta neutrophil index (DNI), coagulation indices (platelets and partial thrombin time), and C-reactive protein (CRP) between survivors and non-survivors (*p* < 0.0001).

### Corrected anion gap as a predictor of mortality in PICU patients

In the univariable analyses, 15 variables were correlated with in-hospital mortality (*p* < 0.01). Of these, 9 variables (age, sex, pH, DNI, platelets, cAG and pre-existing mortality prediction models) were introduced into the multivariable logistic regression analysis. cAG was identified as an independent factor associated with in-hospital mortality [odds ratio (OR) 1.110, 95% confidence interval (CI) 1.06–1.17, *p* < 0.001]. PIM3 and PRISM III were also found to be independently associated with mortality (Table 3).

**Table 3:** Multivariable logistic regression for independent factor in mortality prediction.

	OR	95% CI	*p-*value
Age	1.034	0.97–1.10	0.301
Sex	1.052	0.58–1.93	0.869
pH	0.862	0.06–11.93	0.912
DNI	1.034	1.00–1.10	0.079
Platelets	1.000	0.99–1.00	0.784
cAG	1.110	1.06–1.17	<0.001
PIM2	0.344	0.09–1.39	0.133
PIM3	7.583	1.81–31.78	0.006
PRISM III	1.076	1.02–1.14	0.008

OR, odds ratio; CI, confidence interval; DNI, delta neutrophil index; cAG, corrected anion gap; PIM, Pediatric Index of Mortality; PRISM III, Pediatric Risk of Mortality III.

**Figure 1:**
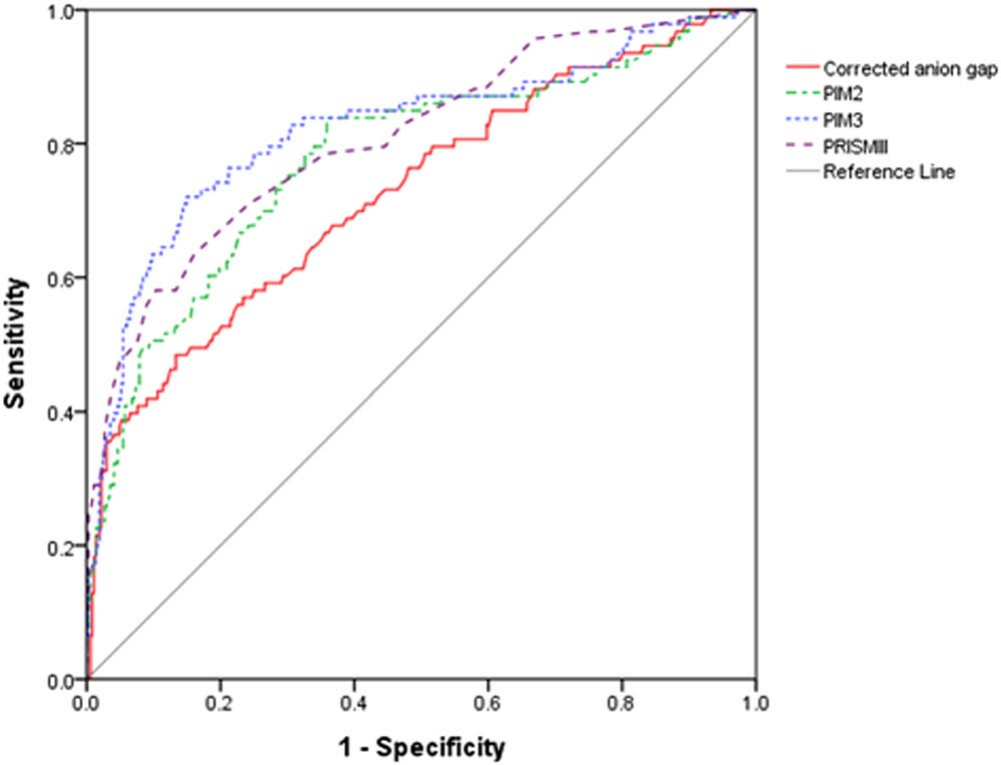
Receiver operating characteristic (ROC) curves for mortality between corrected anion gap and pre-existing mortality prediction models in PICU. There were no differences between corrected anion gap and other prediction models for mortality. PIM3 showed the best power to predict in-hospital mortality (area under the ROC curve, 0.822 [95% CI, 0.767–0.877]).

We also considered relationship between cAG at admission and pre-existing mortality prediction models used in PICU. Each model was significantly correlated with cAG, but the correlation coefficients were relatively low. However, receiver operating characteristic (ROC) curves of cAG, PIM 2, PIM3, and PRISM III showed that there were no differences in mortality prediction. The area under the ROC curve (AUC) was 0.728 for cAG, 0.779 for PIM2, 0.822 for PIM3, and 0.808 for PRISM III score (Table 4, Fig. 1).

**Table 4:** Correlation and AUC comparison between cAG and pre-existing mortality prediction models in PICU.

	Correlation coefficient	*p*-value	AUC (95% CI)	*p*-value
cAG	—	—	0.728 (0.666–0.789)	—
PIM2	0.183	0.0001	0.779 (0.722–0.837)	0.799
PIM3	0.182	0.0001	0.822 (0.767–0.877)	0.074
PRISM III	0.241	<0.0001	0.808 (0.755–0.861)	0.105

AUC, Area under the receiver operating characteristic curve; CI, confidence interval; cAG, corrected anion gap; PICU, pediatric intensive care unit; PIM, Pediatric Index of Mortality; PRISM III, Pediatric Risk of Mortality III.

Correlation is significant at the 0.01 level.

The incorporation of cAG to pre-existing mortality prediction models improved their ability to predict mortality (Table 5). The incorporation of cAG to PIM2 gave an NRI of 17.5% (*p* = 0.001) and IDI of 7.9% (*p* < 0.001). The PIM3 with cAG provided an NRI of 16.7% (*p* = 0.002) and IDI of 7.3% (*p* < 0.001). And the incorporation of cAG to PRISM III yielded an NRI of 9.4% (*p* = 0.043) and IDI of 5.2% (*p* < 0.001). All cAG-incorporated models showed good discrimination but, the models of PIM3 with cAG showed was revealed poor calibration in Hosmer–Lemeshow goodness-of-fit test (*p* = 0.0004) (Supplementary Table S1).

**Table 5:** NRI and IDI for assessing improvement in mortality prediction after incorporating cAG to pre-existing mortality prediction models in PICU.

	NRI		IDI	
	Value (SE)	*p*-value	Value (SE)	*p-*value
PIM2 with cAG	0.175 (0.053)	0.001	0.079 (0.018)	<0.001
PIM3 with cAG	0.167 (0.054)	0.002	0.073 (0.018)	<0.001
PRISMIII with cAG	0.094 (0.046)	0.043	0.052 (0.014)	<0.001

NRI, Net Reclassification Improvement; IDI, Integrated Discrimination Improvement; cAG, corrected anion gap; PICU, pediatric intensive care unit; SE, standard error; PIM, Pediatric Index of Mortality; PRISM III, Pediatric Risk of Mortality III.

**Figure 2:**
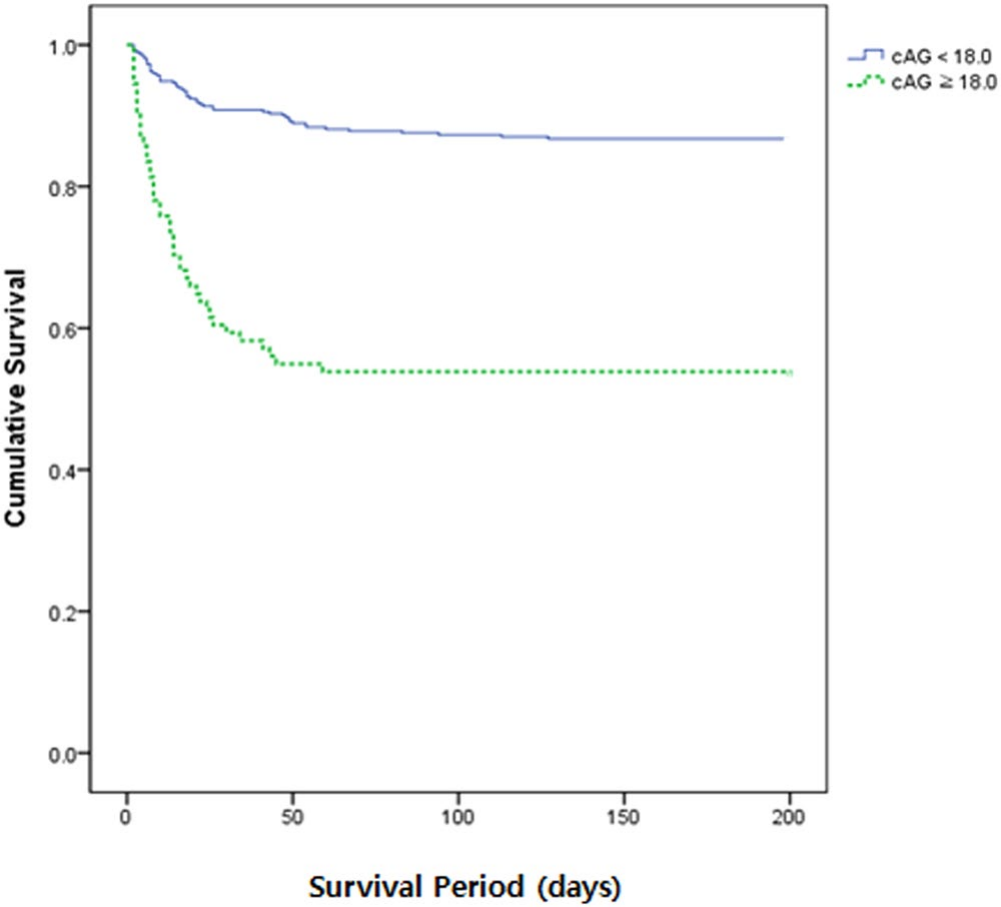
Survival curves for the patients according to cutoff value of corrected anion gap. The cutoff value of initial corrected anion gap was defined by 18.0 mEq/L (*p* < 0.001 by log-rank test).

And comparing AUCs between pre-existing models and cAG-incorporated models, all cAG-incorporated models showed improved performance in mortality prediction. But the model of PIM3 with cAG showed no statistical significance as well in AUC comparison, even though AUC of PIM3 with cAG (0.855) was greater than PIM3 (0.822) (Supplementary Table S2). In this study, the best cutoff value of cAG to predict mortality was 18.0 mEq/L (sensitivity 46.2%, specificity 87.0%). Figure 2 shows survival curves based on data from all 461 patients stratified by cutoff value of cAG (cAG < 18.0 vs. cAG ≥ 18.0 mEq/L, *p* < 0.001 by log-rank test).

## Discussion

In this study, serum cAG calculated at the time of PICU admission was higher in non-survivors than in survivors. Increased cAG was associated with in-hospital mortality, and was an independent predictor of mortality in PICU patients. And cAG was correlated with pre-existing mortality prediction models for children. cAG could be a mortality predictor in critically ill children, regardless of the presence of metabolic acidosis or their underlying etiology.

Acid-base derangements are common in critically ill patients. Although the pathogenesis is not fully understood, it is well-known that ongoing acid-base disequilibrium could reflect the severity of disease and is associated with a poor prognosis of the patient^7^. Many studies have observed a strong association between acidosis and increased organ dysfunction and mortality. However, it remains difficult to ensure accurate measurement of in ICU patients because of their complex and mixed clinical situations of each^8, 9^.

In recent years, quantitative approaches to acid-base disturbances have been increasingly applied to clinical practice. These aim to give information about unmeasured anions or strong ion differences for quantitative evaluation of acid-base derangements in the ICU^10–12^. Previous studies compared traditional biomarkers, such as pH, base excess, or lactate, as means of assessing acid-base disorders and predicting prognosis in critically ill patients^13–16^. However, their reliability has not been established yet. Therefore, reassessing the clinical application of AG, the easiest and most readily available way to calculate acid-base disequilibrium, is useful and meaningful.

AG is a traditional tool used to assess acid-base status and aid the differential diagnosis of metabolic acidosis. Because hypoalbuminemia could affect its interpretation, AG should be corrected for serum albumin level. An elevated cAG usually reflects the presence of metabolic acidosis caused by the overproduction or decreased excretion of organic acids. In addition, elevated cAG has been reported as a predictor of mortality in critically ill patients^5, 6, 10, 11^. In patients with *Streptococcus pneumoniae* bacteremia^5^ and acute myocardial infarction^6^, the presence of an increased cAG acidosis was associated with increased mortality.

Mortality is considered the most reliable endpoint of clinical management in ICU setting. Therefore, estimating mortality risk is an important of ICU care. Several models are available to predict the risk of death of children in PICU. By keeping these models updated, the clinical meaning and validity of each prediction model have been proved. However, with ongoing revision and updating, these models have become more complicated. They require at least 10 variables to be collected from patients, have different coding rules, and each model relies on complex calculations, making their application difficult^1, 17, 18^. Therefore, there is an increasing need to establish an easy clinical method for use in PICU settings. Several biomarkers such as DNI^19^, CRP^20^, procalcitonin^21^, thrombocytopenia^22^ and eosinopenia^23^ have been suggested for use in PICU.

We conducted this study to test whether cAG at admission can predict mortality or morbidity in PICU patients. Comparing survivors and non-survivors, we determined that an increased cAG at admission to PICU was strongly associated with in-hospital mortality, regardless of the underlying etiology. In terms of the multivariable logistic regression analysis for mortality prediction, cAG at admission was identified as the strongest independent factor associated with in-hospital death. Furthermore, incorporation of cAG at admission to pre-existing mortality prediction models improved their ability of to predict mortality in this study. Considering the complex characteristics of physiology and disease etiologies in children, measurement of cAG at admission to PICU could aid understanding of current status and the clinical outcome of pediatric patients. This study shows that cAG, which is a simple method to calculate, does not require arterial puncture and is readily available, could be a marker of mortality prediction in PICU. The limitation of this study is that data were collected retrospectively. We assessed only the initial cAG at admission to PICU, and obtained the results associated with mortality. And considering incorporation of cAG to pre-existing mortality prediction models, the model of PIM3 with cAG showed good discrimination but poor calibration. There was still a clinical significant improvement in mortality prediction, even if not statistical significant when comparing AUCs between PIM3 and cAG-incorporated PIM3. We think that because predictability of standalone PIM3 is already very powerful and the sample size was relatively small. But owing to the results of NRI, IDI, and an increase in AUC from 0.822 of PIM3 to 0.855 of PIM3 with cAG, cAG is still a useful tool to add on to other mortality prediction models in general. Further studies might be needed as a prospective design in a large multicenter setting to validate the ability of cAG to predict clinical outcome. And serial assessment of cAG could provide much more information about prognosis in PICU.

In conclusion, an elevated cAG at admission was associated with higher mortality in PICU. We suggest that cAG at admission may be used to predict mortality in PICU, regardless of underlying etiology. And the incorporation of cAG to pre-existing mortality prediction models could improve their performance.

## Methods

### Study population

This was an observational study using data collected retrospectively from the medical records of children admitted to the PICU at Severance Hospital, Seoul, Korea between December 2009 and February 2015. Patients were over 1 month and under 18 years of age and were in the care of the Department of Pediatrics. Patients were admitted to PICU from the emergency department or the general ward. Patients who were discharged or died within 24 hours of PICU admission were excluded. Neonates, patients with cardiac diseases, and patients requiring post-operative care were admitted to and treated in separate specialized units, and were thus excluded from this study.

### Data collection

All data were collected and analyzed retrospectively. Blood samples were obtained from indwelling arterial catheters or by venipuncture on admission to PICU. The samples were immediately transported to the central laboratory based in the hospital. Initial arterial blood gases, complete blood cell counts, coagulation indices, serum electrolytes, and levels of albumin were analyzed. The AG was calculated as:

1 $$AG=[N{a}^{+}]-([C{l}^{-}]+[tC{O}_{2}])$$

Compensation for abnormal albumin levels was achieving the following equation:

2 $$corrected\,anion\,gap\,(cAG)=AG+{2.5}x\,({4}-albumin(g/dL))$$

Normal cAG values were defined as 3–12 mEq/L, using the ion-specific electrodes method (Hitachi 747 Manual; Roche Diagnostics, Sydney, NSW, Australia)^8^. Metabolic acidosis was defined as a standard base excess (SBE) < −2 mEq/L, using following formula^7^:

3 $$SBE={0.928}x\,(HC{O}_{3}-{24.4}+{14.83}[pH-{7.4}])$$

Age, sex, in-hospital mortality, length of stay in PICU, underlying etiology, reasons for admission, and requirement for mechanical ventilation within 24 hours of PICU admission were recorded. Concomitant metabolic problems that could affect acid-base status and cAG, such as liver or renal failure, were also recorded. For all patients, Pediatric Index of Mortality (PIM) 2^18^ and PIM 3^1^ were recorded at admission, and the Pediatric Risk of Mortality III (PRISM III)^17^ was recorded at 24 hours after admission to PICU. PIM and PRISM III scores were calculated on the basis of required variables of each. And then PIM2 and PIM3 were expressed as ‘logit_probability’ based on the logistic regression equation for the derived scores^17^. But PRISM III was expressed as calculated scores^24^.

### Statistical analysis

Baseline characteristics of patients were compared using Mann-Whitney U test or Fisher’s exact test, as appropriate. Data were reported as numbers (percentages) or medians (inter-quartile range). Groups were compared by the chi-square test or Fisher’s exact test for categorical variables, and the Mann–Whitney *U* test, as appropriate. Among variables with *P* < 0.01 in the univariable logistic regression analyses, less than 9 variables without missing values were introduced into multivariable logistic regression analysis. Multivariable logistic regression model was used to identify independent predictors of mortality and to examine the relation between cAG and mortality. The correlation between cAG and pre-existing mortality prediction models was assessed using Spearman’s method. Survival curves were determined using Kaplan-Meyer method, and the differences in survival according to cutoff values of cAG were analyzed by means of the log-rank test. The optimal cutoff value of cAG to predict mortality was chosen by the Youden index and the AUC. Statistical analyses were performed with SPSS 20.0 (SPSS Inc., Chicago, IL).

The predictive values of cAG and PIM2, PIM3, PRISM III for predicting in-hospital mortality were compared by calculating the AUC. Between pre-existing models and cAG-incorporated models (PIM2 with cAG, PIM3 with cAG, and PRISMIII with cAG), Hosmer-Lemeshow goodness-of-fit test for calibration to assess applicability and ROC curve for discrimination and comparisons of AUCs were used by SAS 9.4 (SAS Inc., Cary, NC).

And the net reclassification improvement (NRI) and integrated discrimination improvement (IDI) statistics are introduced to assess the incremental predictive impact of new models that integrate a candidate marker (cAG) to pre-existing models (PIM2, PIM3 or PRISM III)^25^. Subjects are considered separately who develop and who do not develop events; patients who are dead or survived in this case. For NRI, each subject is assigned to a risk category based on the death probability calculated by the pre-existing mortality prediction models. And then each subject is reassigned to a risk category using new mortality prediction models, constructed by incorporation of cAG to pre-existing mortality prediction models. After calculating the net proportion of subjects with death reassigned to a higher risk category (NRI_evemt_) and of subjects without events reassigned to a lower risk category (NRI_nonevent_), the NRI is the sum of NRI_event_ and NRI_nonevent_. And the IDI considers separately the actual change in calculated risk for each individual for those with and those without events. The IDI_event_ is the difference between the mean probability of a new mortality prediction model for those with the event and the mean probability of the existing models for those with the event. The IDI for those without events (IDI_nonevent_) is the difference in mean probability for those who do not have the event between the reference and new models. The IDI is expressed as the total of IDI_events_ and IDI_nonevents_. If the value of NRI and IDI are over 0, it means that the performance of new models to predict mortality is improved than existing models in sensitivity and specificity^26^. NRI and IDI were performed using R software (R version 3.0.1). *P* < 0.05 was considered statistically significant.

### Ethics statement

This study was approved by the institutional review board of Severance Hospital [Seoul, Korea, IRB No. 4-2012-0369]. All protocols and methods in this study were carried out in accordance with relevant guidelines and regulations. And this study was exempted from the informed consent due to retrospective observational study followed by the institutional review board of Severance Hospital.

## Electronic supplementary material

Supplemet Tables
