# Supplementary material for: Serum anion gap at admission as a predictor of mortality in the pediatric intensive care unit
Source: Sci Rep. 2017 May 3;7:1456. doi: 10.1038/s41598-017-01681-9 (PMC5431089; doi:10.1038/s41598-017-01681-9)
Supplement: Supplementary file 1 — Supplemet Tables [file 41598_2017_1681_MOESM1_ESM.pdf]

Serum anion gap at admission as a predictor of mortality in the pediatric intensive care unit

[1]Min Jung Kim, MD; [1]Yoon Hee Kim, MD; [1]In Suk Sol, MD; [1]Soo Yeon Kim, MD; [1]Jong Deok Kim, MD; [2]Ha Yan Kim, MS; [1]Kyung Won Kim, MD, PhD; [1]Myung Hyun Sohn, MD, PhD; [1]Kyu-Earn Kim, MD, PhD

[1]Department of Pediatrics, Severance Hospital, Institute of Allergy, Brain Korea 21 PLUS Project for Medical Science, Yonsei University College of Medicine, Seoul, Korea
[2]Biostatistics Collaboration Unit, Yonsei University College of Medicine, Seoul, Korea

Table S1. Calibration and discrimination to evaluate performance of each incorporated models

	HL χ^2 (df)	HL p-value	AUC (95% CI)
**PIM2 with cAG**	13.9429 (8)	0.0833	0.836 (0.786-0.885)
**PIM3 with cAG**	28.617 (8)	0.0004	0.855 (0.808-0.902)
**PRISM III with cAG**	3.4018 (8)	0.9067	0.834 (0.785-0.883)

HL, Hosmer and Lemeshow Goodness of fit test; χ^2, chi-square; df, degree of freedom; AUC, area under the receiver operating characteristic curve; CI, confidence interval; cAG, corrected anion gap; PIM, Pediatric Index of Mortality; PRISM III, Pediatric Risk of Mortality III.

Table S2. AUC comparisons between pre-existing models and cAG-incorporated models

	AUC (95% CI)	p-value
**PIM2**	0.779 (0.722-0.837)	0.0047
**PIM2 with cAG**	0.836 (0.786-0.885)	
**PIM3**	0.822 (0.766-0.877)	0.1151
**PIM3 with cAG**	0.855 (0.808-0.902)	
**PRISM III**	0.808 (0.55-0.861)	0.0385
**PRISM III with cAG**	0.834 (0.785-0.883)	

AUC, area under the receiver operating characteristic curve; CI, confidence interval; cAG, corrected anion gap; PIM, Pediatric Index of Mortality; PRISM III, Pediatric Risk of Mortality III.
